# Primary care physicians and infant mortality: Evidence from Brazil

**DOI:** 10.1371/journal.pone.0217614

**Published:** 2019-05-31

**Authors:** Letícia Xander Russo, Anthony Scott, Peter Sivey, Joilson Dias

**Affiliations:** 1 Department of Economics, State University of Maringá, Maringá Paraná, Brazil; 2 Melbourne Institute: Applied Economic and Social Research, The University of Melbourne, Melbourne, Victoria, Australia; 3 School of Economics Finance and Marketing, RMIT University, Melbourne, Victoria, Australia; Aga Khan University, KENYA

## Abstract

Primary health care has been recognized as a critical strategy for improving population health in developing countries. This paper investigates the effect of primary care physicians on the infant mortality rate in Brazil using a dynamic panel data approach. This method accounts for the endogeneity problem and the persistence of infant mortality over time. The empirical analysis uses an eight-year panel of municipalities between 2005 and 2012. The results indicate that primary care physician supply contributed to the decline of infant mortality in Brazil. An increase of one primary care physician per 10,000 population was associated with 7.08 fewer infant deaths per 10,000 live births. This suggests that, in addition to other determinants, primary care physicians can play an important role in accounting for the reduction of infant mortality rates.

## Introduction

Health care systems with a strong primary care orientation are more likely to provide better population health and more equitable health outcomes [1]. Primary care is generally defined as dealing with a wide range of health problems in the community, health promotion, disease prevention and rehabilitation as set out in Alma-Ata [2]. Although studies show the importance of primary care to health outcomes, evidence of the effect of changes in the level of human resources for health is limited. The majority of previous studies investigating the association between primary care physicians and health outcomes are cross-sectional and derived from sources that do not allow testing of the effect of changes in primary care on changes in outcomes over time. To our knowledge, there is only two longitudinal study that analyzed the relationship between primary care physicians (PCPs) and mortality rates at disaggregated level over time. Basu et al. [3] found that an increase in primary care physician density was associated with lower mortality in the United States. Greater PCP density showed a higher effect on life expectancy than specialist physician density, 51.5 day and 19.2 day increase in life expectancy, respectively. Aakvik and Holmas [4] did not find a statistically significant relationship between the level of PCP supply and mortality in Norway. However, Norway has less inequality than the United States and Brazil, and so there may be little room for improvement.

Using longitudinal data, but at state level, Shi et al. [5] found that increased PCP supply led to reductions in infant mortality in US. They investigated the relationship between primary care and infant mortality in 50 US states for eleven years (1985–95) and control for Gini coefficient, racial composition, rural areas, unemployment and education.

In this paper, we investigate the effect of changes in primary care physician supply on improvements in infant mortality rates (IMR) in Brazilian municipalities between 2005 and 2012. Infant mortality is one of the most used indicators of population health. Moreover, infant mortality is sensitive to a country’s health care system as well as socioeconomic conditions [6].

There are some particular channels through which primary care may influence infant mortality during both the neonatal and post-neonatal period. In 2012, neonatal mortality (deaths between 0–27 days) represented 69.3% of overall infant mortality (under-1 year) in Brazil. Neonatal deaths are associated with the quality of prenatal care [7,8], which allows early identification and intervention to minimize damage to child health. Quality of prenatal care is influenced by access to health care facilities, numbers of prenatal consultations and qualified health workers including physicians [9].

Post-neonatal mortality (deaths between 28–364 days) for ambulatory care-sensitive conditions (ACSC) corresponded to 22% of all post-neonatal mortality in 2012, with a higher incidence of lobar pneumonia, intestinal infectious, acute bronchiolitis and malnutrition (DATASUS/Brazilian Ministry of Health). Shi et al. [5] pointed out two mechanisms through which primary care is associated with lower post-neonatal mortality. First, primary care can improve mother’s and child’s health and better manage conditions affecting post-neonatal age (for example, by providing information about infections and safety). Second, primary care can deal with a series of maternal risk factors linked to infant deaths, such as smoking, alcoholism, poor weight gain during pregnancy, sexually transmitted diseases and poor nutrition.

In Brazil, as in other developing countries, primary health care has been recognized as a central strategy for improving population health, as well as a key instrument to reduce child mortality and achieve the fourth of the Millennium Development Goals (MDG) set by United Nations. The MDG-4 aimed to reduce the under-5 mortality rate by two-thirds for the period from 1990 to 2015. Though the global goal for child mortality rate was not reached, significant achievement has been made in the world (global under-5 mortality has declined more than half) [10].

Brazil showed substantial progress in reducing child mortality over the period, falling by 73 per cent from 1990 to 2015. The number of under-5 deaths was 60.8 deaths per 1,000 live births in 1990 and 16.4 deaths per 1,000 live births in 2015 [11]. However, despite the reduction in the infant mortality rate in Brazil, the country still has relatively high rates of infant deaths, more than 2.4 times when compared with the average for high income countries.

We contribute to the literature through our innovative use of data on primary care physicians from the Brazilian Ministry of Health, which provides annual data on human resources for health for all municipalities since 2005, and a robust methodological approach, dynamic panel data analysis. Dynamic panel data models have also been used in previous studies for analyzing the determinants of infant mortality [12,13]. This method accounts for endogeneity and unobserved municipality-specific effects. Endogeneity may lead to an inconsistent estimator and generate misleading results [14]. This issue has been addressed by only few studies investigating the association between primary care and health outcomes [4,15,16]. In addition, the dynamic panel model improves the accuracy of estimates when the dependent variable is persistent over time. In our case, such persistence could be due to health habits, mother’s behaviours, and unobserved characteristics of the environment which are associated with child health. Furthermore, previous findings of inter-family heterogeneity that drives a positive correlation in infant mortality across siblings [17] suggest that a dynamic model is appropriate.

Our findings from the dynamic panel model demonstrate the effect of primary care physician supply on reducing infant mortality, even after including a range of important determinants of child health. The estimated results strengthen the case for the role of primary care in improving health in developing countries.

## Primary care in Brazil

The Brazilian health system (Unified Health System—SUS) was founded in 1988 on the principles of universality and equality, as well as decentralized management and popular participation [18]. The SUS is funded primarily through taxes and offers free health services, from medical consultations to organ transplants.

In the 1990s, after the creation of SUS, efforts focused on universal access to primary care. The government adopted measures aimed at strengthening primary care incorporating principles such as first contact, continuity, comprehensiveness and coordination—as outlined by Starfield [19]. The implementation of the Family Health Program (now called Family Health Strategy—FHS) by the Ministry of Health in 1994, was a strategy to reorganize health care and establish a new dynamic in the organization of health services and preventive services [20].

Although a broad expansion of the FHS has occurred, the traditional model of primary care still remains. Physicians and staff in both settings are salaried and funded by government, but the models differ in the organization of care, in the composition of the teams and territorial definition of coverage areas.

Traditional healthcare centers include physicians (general practitioners, pediatricians and obstetrician-gynecologists), nurses, nurse assistants and dentists who respond reactively to patient demand. FHS units are composed of a team as specified by the Ministry of Health comprising a primary care physician, nurse, nurse assistant, community health workers, and sometimes dental health professionals. FHS teams are organized geographically and they are guided by the principles of responsibility for the health of the local population through proactive needs assessment. They are designed to bring primary care closer to the communities, including periodic visits to households and consultations at home as well as in healthcare centers [21]. The FHS is a model focused on preventive health care with a multi-professional team, while the traditional model prioritizes curative and individual measures. Currently, primary care is provided by both the FHS and traditional models.

## Data and methods

### Measures

The definition of primary care physicians can be broad and in the US can include family and general practitioners, general internists, and general pediatricians [22]. In this paper, primary care physicians are measured using the sum of the hours worked per week of FHS physicians, community physicians, general practitioners (GPs), general pediatricians and gynecologists/obstetricians who work in healthcare centers (healthcare centers include *Centro de Saúde/Unidade Básica de Saúde*, *Posto de Saúde* and *Centro de Apoio a Saúde da Família—CASF*). Healthcare centers are designed to provide primary health care to the population and they concentrate primary health teams. Physicians working in hospitals are not included. Our measure of primary care physician density is defined as the total number of PCPs per 10,000 population in each municipality. The total number of PCPs is calculated by dividing the total hours worked per week by 40 to arrive at a “full-time equivalent” measure of PCP supply. This measure allows more accuracy to compare the availability of physicians among municipalities, since physicians have diverse workloads in Brazil.

Based on previous literature [23,24], a set of variables known to be associated with infant mortality was included in the analyses as covariates: other FHS team members per 10,000 population, hospital beds per 10,000 population (public and private), percentage of population with supplemental health insurance (private), percentage of female illiteracy, Gross Domestic Product (GDP) per capita and four measures of household facilities (percentage of household with trash collection, available electricity, access to piped water and percentage of household with sewage).

The data are obtained from several databases. Infant mortality and hospital beds are from the DATASUS database at the Brazilian Ministry of Health. Primary care physician and other FHS team are collected from TabWin—program developed by DATASUS/Ministry of Health. Data on supplemental health insurance is taken from National Regulatory Agency for Private Health Insurance and Plans (ANS). Data on GDP and population are obtained from the Brazilian Institute of Geography and Statistics (IBGE). Data on household facilities (trash collection, sewage, available electricity and access to piper water) are taken from National Census Bureau. The data on female illiteracy is based on the Annual List of Social Information (RAIS) from the Ministry for Labor and Employment (MTE); this data is only available for female workers in the formal sector. All data were extracted from publicly sources from links listed in S1 Appendix. The data are also available from authors on request.

Other FHS team members include nurses and nurse assistants. Nurses of FHS are included as covariates based on their relevant role for primary care and infant health [25,26]. The total number of other FHS team members is calculated by dividing the total hours worked per week by 40.

Linear interpolation is used to estimate annual values of the four household facilities variables for the period 2005 to 2009. The estimates are based on the National Census (2000 and 2010). For the posterior period, 2011 and 2012, linear extrapolation is used based on the same function. This technique has been utilized to deal with the lack of annual data from Census [15,27]. Final results are stable when these variables are excluded. Annual values for all other variables are available from the database.

### Descriptive statistics and data

Descriptive statistics of each variable over the whole period are shown in Table 1.

**Table 1:** Descriptive statistics for Brazilian municipalities (2005–2012).

	Obs	Mean	Std. Dev.	Min	Max
Infant mortality rate per 10000 live births	44504	156.43	144.73	0	4285.71
Primary care physician per 10000 population	44504	3.40	1.93	0	32.15
Traditional physician per 10000 pop.	44504	0.65	1.15	0	23.39
FHS physician per 10000 pop.	44504	2.75	1.62	0	31.31
Other FHS team per 10000 population	44504	6.76	4.09	0	52.19
Hospital beds per 10000 population	44504	18.90	22.55	0	404.26
Supplemental Health Insurance (%)	44504	6.79	10.31	0	100
Female illiteracy (%)	44504	0.55	1.84	0	67.27
GDPpc	44504	13425	15228	1513	511967
Trash collection (%)	44504	66.43	23.55	0	100
Electricity (%)	44504	93.88	12.75	0	100
Piped water (%)	44504	81.16	21.76	0	100
Sewage (%)	44504	28.97	30.53	0	100

Table 2 reports the means of the variables for each time period. The infant mortality rate decreased 20% over period, while all of the household facilities recorded an improvement over the entire period. The number of primary care physicians per 10,000 population increased from 3.0 in 2005 to 3.7 in 2012. After the implementation of FHS in 1994 the number of FHS physicians and other FHS team members has increased, while the number of physicians of traditional models has decreased over the period.

**Table 2:** Variable means for Brazilian municipalities, by year (2005–2012).

	2005	2006	2007	2008	2009	2010	2011	2012
Infant mortality rate	175	175	165	158	153	143	143	140
Primary care physician per 10000 pop.	3.0	3.0	3.3	3.5	3.5	3.6	3.6	3.7
Traditional physician per 10000 pop.	0.8	0.8	0.7	0.7	0.6	0.6	0.6	0.6
FHS physician per 10000 pop.	2.2	2.2	2.6	2.8	2.9	3.0	3.0	3.1
Other FHS team per 10000 pop.	5.1	5.4	6.4	6.8	7.2	7.5	7.7	8.0
Hospital beds per 10000 pop.	20.4	20.2	19.9	18.6	18.4	18.3	17.9	17.5
Supplemental Health Insurance (%)	5.2	5.9	6.1	6.6	7.0	7.6	8.0	8.3
Female illiteracy (%)	0.9	0.8	0.7	0.6	0.5	0.5	0.2	0.2
GDPpc	11,336	11,999	12,493	12,917	13,915	14,510	15,336	20,685
Trash collection (%)	60	62	64	66	67	69	71	72
Electricity (%)	90	91	92	93	94	96	96	97
Piped water (%)	75	77	79	80	82	84	85	86
Sewage (%)	27	28	28	29	29	30	30	31

We use data from the Brazilian Ministry of Health (DATASUS—Department of the SUS), and follow a balanced panel of 5,563 municipalities in Brazil over a period of 8 years from 2005–2012 for the main analysis. Data on PCPs was available at municipal level only after 2005.

In addition, we performed sensitivity analyses for alternative samples. The reason for these alternative estimations is that the quality of mortality data may vary across regions of Brazil because of inadequate death registration systems, especially in the North and Northeast regions. These regional differences in death reporting may lead to the underestimation of the infant mortality rate especially in poorer areas and those with limited access to health care. These issues may not be as concerning for longitudinal data analyses. Nevertheless, the model estimates may be affected by improvements in the reporting system over time though this would be captured by the year fixed effects [28]. To ensure that the results from the main model are consistent and valid, we employed a method to reduce measurement error in the municipality-level mortality data. This method consists of analyzing the quality of information on births and deaths for all Brazilian municipalities and including only those municipalities who met a previously criteria defined by Frias et al [28].

Frias et al. [28] analyzed the adequacy of the reporting system using municipal indicators constructed with data from Ministry of Health and developed a method for selecting data from only the most reliable municipalities which has since been used by previous studies [27,29]. The Frias et al. [28] method validates the information about births and deaths at municipality level using five indicators. *i)* Age standardized mortality rate: values above 5 per 1,000 inhabitants indicate adequate death reporting. *ii)* Average annual deviation from the three-year period mean of the age standardized mortality rate: deviations of less than 20% indicate reliable information. *iii)* Ratio of reported live births to estimated live births: rates above 0.85 represent reliable information. *iv)* Average annual deviation from the three-year period mean of the birth rate: deviations below 20% indicate it was adequately reported. *v)* Proportion of ill-defined deaths (the use of imprecise terms and dubious expressions that prevent the identification and coding of cause of death): values below 20.7% for municipalities with population of less than 50,000 and values below 16.2% for municipalities with population of more than 50,000 indicate adequate death reporting [30].

The sensitivity analyses were performed for two alternative estimation samples: (A) Frias et al. [28] criteria in a balanced panel (5,110 observations from 730 municipalities) and (B) Frias et al. [28] criteria in an unbalanced panel (14,095 observations from 3,850 municipalities).

### Method

The empirical analysis of this study uses the System Generalized Method of Moments (GMM) estimator developed by Arellano and Bover [31] and Blundell and Bond [32] for dynamic models. Consider the dynamic equation represented below

$${im}_{it}={\delta im}_{it-1}+{\gamma PCP}_{it}+\beta^{\prime }X_{it}+\lambda *year+\eta_{i}+\varepsilon_{it}$$ (1)

where *im*_*it*_ is the infant mortality rate of municipality *i* at year *t*; *im*_*it*-1_ denotes the lagged infant mortality rate and it is included to capture persistence in infant mortality; *PCP*_*it*_ is the density of primary care physicians; *X*_*it*_ contains a set of explanatory variables; *λ* is the time trend in the infant mortality rate of all municipalities; *η*_*i*_ is the unobserved municipality-specific effect; and *ε*_*it*_ denotes the error term.

The estimation of Eq (1) using pooled OLS will potentially lead to biased and inconsistent results as the orthogonality condition is violated [33]. This is a well-known problem of pooled OLS, as the correlation between *η*_*i*_ and *im*_*it*−1_ or *X*_*it*_ is very likely to be non-zero. The fixed effects model accounts for the municipality-specific effects but requires an assumption of strict exogeneity, which never holds in models with a lagged dependent variable [14].

We follow Arellano and Bond [34], who suggest an estimator using the Generalized Method of Moments that includes the lagged levels of the explanatory variables as instruments. To eliminate the fixed municipality specific effect, differenced-GMM estimates the Eq (1) in first differences

$${\Delta im}_{it}={\delta \Delta im}_{it-1}+{\gamma \Delta PCP}_{it}+\beta^{\prime }\Delta X_{it}+\Delta \lambda *year+{\Delta \varepsilon }_{it}$$ (2)

where Δ*im*_*it*_ = *im*_*it*_ − *im*_*it*−1_. Since Δ*im*_*it*−1_ and Δ*ε*_*it*_ are correlated by definition, the OLS estimate is inconsistent, hence the need for an instrumental variables approach. Blundell and Bond [32] pointed out that the GMM estimator obtained after first differencing is likely to perform poorly when the number of time periods is moderately small and the autoregressive parameter is moderately large. In such a case lagged levels of the series provide weak instruments for first differences. To cope with this problem, Arellano and Bover (1995) and Blundell and Bond [32] proposed a system of regressions in differences and levels, where the instruments used for the levels are lagged first-differences of the explanatory variables. System GMM can handle endogenous regressors, fixed individual effects and unobservable heteroskedasticity [35].

One concern in using System GMM is instrument proliferation. The number of instruments tends to easily grow with the number of time periods, making some asymptotic results and weakened specification tests. We follow the approach of Roodman [36], who suggests that the instrumental variables can be reduced by collapsing instruments and limiting lag depth amounts.

## Results

Eq (1) is estimated with four different estimation methods (pooled OLS, random effects, fixed effects and System GMM). The regressions include a full set of covariates informed by the previous literature and control for time trends. All regressions use robust standard errors.

S2 Appendix reports the results of pooled OLS, random effects and fixed effects (within). Our estimates consistently show the lagged dependent variable (the infant mortality rate) is statistically significant, indicating a low degree of persistence in the infant mortality. Column (1) and (2) show the results of pooled OLS, column (3) and (4) report the results of random effects and column (5) and (6) the results of fixed effects. The coefficient on the number of PCPs is statistically significant using all estimation methods, except using fixed effects estimation after including time trends (column (6)).

However, as discussed previously and addressed by Nickell (1981), in dynamic panel data models, fixed effects estimation, as well as pooled OLS and random effects, may be biased and inconsistent. To deal with endogenous regressors, among other concerns, the Eq (1) is estimated using System GMM.

Table 3 reports the System GMM estimations. The table presents different specifications to test the sensitivity of the results to including additional controls. The Hansen/Sargan test of over-identifying restrictions show the null hypothesis cannot be rejected in the System GMM, consistent with the hypothesis that the instruments are valid for the estimation. In addition, we also test for second-order residual serial correlation and there is no evidence for significant AR(2) serial correlation of the residuals. To correct for downwards biased standard errors in finite samples, we employ a System GMM two-step estimator implementing the correction developed by Windmeijer [37]. In addition, we follow an empirical strategy to deal with instrument proliferation in the System GMM. Following the approach of Roodman [35] we collapsed the instruments and restricted the number of lags used in the model.

**Table 3:** Estimation results for infant mortality using System GMM.

	(1)	(2)	(3)	(4)	(5)	(6)	(7)	(8)	(9)
Infant Mortality_t-1_	0.0199*	0.0206*	0.0207*	0.0208*	0.0204*	0.0200*	0.0199*	0.0208*	0.0200*
	(0.0109)	(0.0109)	(0.0109)	(0.0109)	(0.0109)	(0.0110)	(0.0110)	(0.0109)	**(0.0110)**
PC Physicians	-5.479**	-6.937**	-6.917**	-6.960**	-7.212***	-6.591**	-6.940**	-6.957**	-7.082***
	(2.577)	(2.743)	(2.729)	(2.729)	(2.706)	(2.730)	(2.699)	(2.729)	**(2.706)**
Other FHS team		1.405	0.818	0.887	0.533	0.668	0.642	0.912	**0.634**
		(1.080)	(1.117)	(1.116)	(1.119)	(1.110)	(1.102)	(1.126)	**(1.114)**
Hospitalbeds			-0.119***	-0.114**	-0.0616	-0.0789*	-0.0536	-0.114**	**-0.0347**
			(0.0456)	(0.0455)	(0.0465)	(0.0471)	(0.0481)	(0.0455)	**(0.0475)**
Supplemental Health Insurance (%)			-1.283***	-1.043***	-0.665***	-0.912***	-0.740***	-1.095***	-0.874***
			(0.133)	(0.153)	(0.160)	(0.161)	(0.169)	(0.153)	**(0.150)**
Female illiteracy (%)				2.743**	2.726*	2.780**	2.317*	2.714**	2.207*
				(1.385)	(1.411)	(1.384)	(1.326)	(1.383)	**(1.330)**
Gdppc				-0.0004***	-0.0003***	-0.0004***	-0.0003***	-0.0004***	-0.0002***
				(0.0001)	(0.0001)	(0.0001)	(0.0001)	(0.0001)	**(0.0001)**
Trash coll. (%)					-0.420***				-0.199***
					(0.0582)				**(0.0767)**
Electricity (%)						-0.613***			**-0.0242**
						(0.181)			**(0.204)**
Piped water (%)							-0.542***		-0.480***
							(0.0870)		**(0.0949)**
Sewage (%)								0.0370	0.201***
								(0.0365)	**(0.0386)**
Year	-5.250***	-5.686***	-4.863***	-4.538***	-3.813***	-3.954***	-3.765***	-4.549***	-3.539***
	(0.456)	(0.547)	(0.599)	(0.607)	(0.609)	(0.606)	(0.578)	(0.609)	**(0.597)**
Constant	10,717***	11,587***	9,950***	9,297***	7,868***	8,182***	7,787***	9,320***	7,339***
	(910.6)	(1,092)	(1,195)	(1,212)	(1,216)	(1,208)	(1,156)	(1,217)	**(1,190)**
Observations	38,938	38,938	38,938	38,938	38,938	38,938	38,938	38,938	**38,938**
N of municipalities	5,563	5,563	5,563	5,563	5,563	5,563	5,563	5,563	**5,563**
Instruments	12	15	17	19	20	20	20	20	**23**
Hansen/Sargan test	12.02	16.13*	13.88	13.58	15.58	12.41	11.89	13.59	**11.75**
AR1 test	-25.3	-25.32***	-25.30***	-25.19***	-25.18***	-24.89***	-24.97***	-25.29***	-24.91***
AR2 test	1.15	1.2	1.19	1.19	1.14	1.08	1.08	1.19	**1.08**
Wald Chi2	301.84	310.12	576.71***	623.35***	709.09***	643.05***	721.89***	620.52***	853.96***

Standard errors in parentheses

*** p<0.01,

** p<0.05,

* p<0.1

All specifications of the model find a negative and statistically significant coefficient on PCPs of a similar order of magnitude (ranging from -5.479 to -7.082). The first column shows the regression results including only PCP and time trends. Column (2) includes other FHS team, column (3) adds hospital beds and supplemental health insurance, column (4) contains female illiteracy and GDP per capita.

The columns 5–8 include household facilities separately. The trash collection, available electricity and piped water coefficients are negative and statistically significant. This indicates the negative association between household facilities and the infant mortality rate. The statistical insignificance of the electricity coefficient and the positive effect of sewage coefficient in column (9), which included all covariates, may be related to the presence of multicollinearity between the household variables.

In the most complete specification (column (9)), an increase of one primary care physician per 10,000 is associated with a 7.08 fewer infant deaths per 10,000 live births or 4.5% of the mean infant mortality rate.

In order to test the individual effect of the traditional and FHS models of primary care, the main specification, with all controls, was estimated considering PCPs in both models separately (S3 Appendix). The results show that only the coefficient on FHS physicians (-10.05) was statistically significant.

In addition, we test for a non-linear relationship between PCPs and infant mortality, where the effect of PCPs may at first increase and then decline with each additional PCP. The main model was re-estimated including a squared term and both variables were found to statistically significant with a U-shaped relationship (Table 4).

**Table 4:** Estimation results for a nonlinear specification.

	(1)
Infant Mortality_t-1_	0.0208*
	(0.0109)
PC Physicians	-13.02*
	(7.000)
PC Physicians squared	0.973*
	(0.585)
Year	Yes
Additional controls	Yes
Observations	38,938
N of municipalities	5,563
Instruments	28
Hansen/Sargan test	28.73
AR1 test	-25.06***
AR2 test	1.01
Wald Chi2	873.63***

Standard errors in parentheses

*** p<0.01,

** p<0.05,

* p<0.1

**Fig 1:**
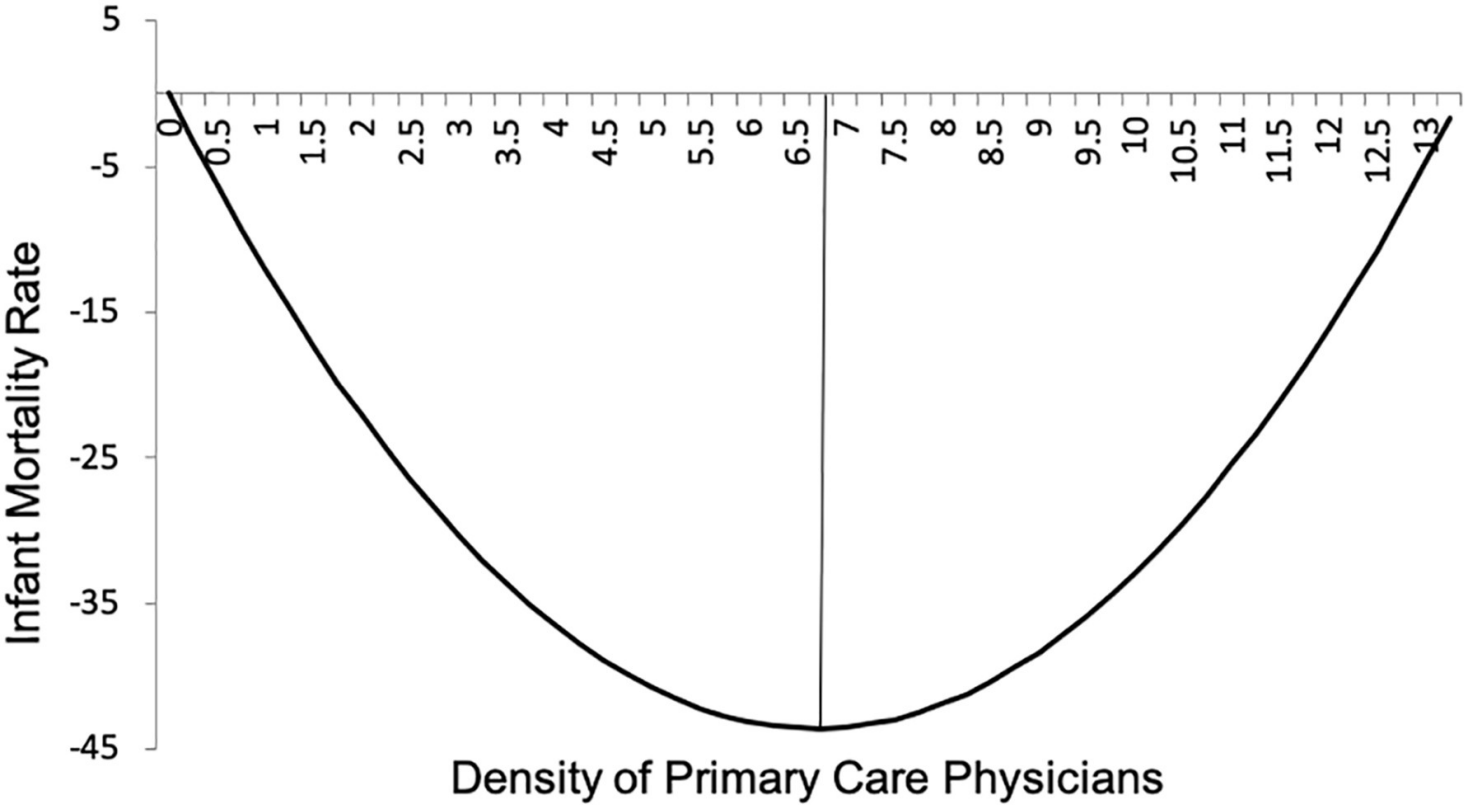
Non-linear relationship between infant mortality rate and density of primary care physicians.

Based on these results (Table 4), we can estimate the approximate level of primary care physicians that minimizes infant mortality rates. According to our data the turning point is approximately at 6.7 PCPs per 10,000 population. The mean value across municipalities in 2012 was 3.4 PCPs per 10,000 population (S.D 1.93), suggesting that in the range of the estimation sample further increases in the number of PCPs could continue to reduce infant mortality but with diminishing returns to the supply of PCPs. The predicted relationship between the density of primary care physicians and infant mortality is shown in Fig 1.

A placebo test was also conducted using the mortality rate for all ages from external causes (ICD-10 codess V00-Y99) as an alternative dependent variable. External causes include mortality by transport accident, falls, assault, drowning, among others and hence, the number of primary care physicians should have no impact as there is no plausible mechanism through which PCPs could influence this measure of mortality. The coefficient on PCPs is found to be near zero and statistically insignificant (S4 Appendix). This result supports the validity of our empirical strategy.

Finally, since the results are based on all Brazilian municipalities, we performed a sensitivity analysis. Table 5 reports the results for the main model (column (9) in Table 3) using alternative estimation samples. Besides the full sample, Eq (1) was estimated using two different estimation samples: Frias et al. [28] criteria in a balanced panel (column A) and Frias et al. [28] criteria in an unbalanced panel (column B). The coefficient of primary care physicians was found to be statistically significant, but to have a larger order of magnitude in both samples. The other FHS team members coefficient also had a larger magnitude in these sub-samples when compared with the full sample. A larger magnitude of the coefficients could be explained by inadequate records of deaths and births over the period in the full sample; this follows from the method of selecting the restricted sample which involves excluding municipalities with a high standard deviation in mortality rates and municipalities with very low reported mortality rates. Although with a variation in magnitude, these results confirm that the negative effect of primary care physicians on infant mortality is found in all samples.

**Table 5:** Estimation results for sensitivity analyses on alternative estimation samples.

	(A): Restricted Sample: Balanced Panel	(B): Restricted Sample: Unbalanced Panel
Infant Mortality_t-1_	0.370**	0.467***
	(0.177)	(0.172)
PC Physicians	-16.20*	-14.74*
	(9.371)	(8.808)
Other FHS team	6.448*	1.698
	(3.369)	(3.300)
Year	Yes	Yes
Additional controls	Yes	Yes
Observations	5,110	14,095
N of municipalities	730	3,850
Instruments	37	37
Hansen/Sargan test	31.4	33.18
AR1 test	-4.05***	-4.88***
AR2 test	1.1	2.42**
Wald Chi2	490.98	1752.45

Standard errors in parentheses

*** p<0.01,

** p<0.05,

* p<0.1

## Discussion

This study assesses the relationship between primary care physicians and infant mortality at the municipal level in Brazil using panel data from 2005 to 2012. Our findings support the results of previous studies which have emphasized the role of primary care in improving health in developing countries while addressing concerns that much of the previous literature suffer, such as their weak methodological approach, including inadequate covariates, and the lack of longitudinal sources which allows to evaluate the effect of primary care on outcomes over time [38]. In this study, we control for a wide range of covariates and use a robust dynamic panel model to account for the endogeneity problem.

Our results show a negative effect of the density of primary care physicians on infant mortality in Brazil. In particular, our results are consistent with two similar previous studies from Brazil but using data from an earlier time period, though we find larger effects. Sousa et al. [9] used data between 1991 and 2000, of 4,267 Minimum Comparable Areas (MCA), and found that an increase of one physician (including non-PCPs) per 1,000 population reduces neonatal mortality by 2.3%. Macinko et al. [39], using microregional data from 1999 to 2004, found that a 10% increase in population coverage of the FHS reduced infant mortality by 0.45%. Our study finds an increase of one PCP per 10,000 reduces infant mortality by 7.08 per 10,000 live births, or 4.5% of the mean infant mortality rate. Extrapolating our results, an increase of 10% in primary care physicians (0.37 PCPs per 10,000 population) predicts a reduction in infant deaths by 765 per year. Sensitivity analysis, which includes only those municipalities with high quality of information [28], shows that this effect can be even stronger.

In addition, two other studies, using different methods, also support our results. Rocha & Soares [40], using a difference-in-difference estimator and data from 1994 to 2004, found that municipalities which implemented the FHS for three years were associated with a 6.5% reduction in IMR and 20% reduction in municipalities with 8 year of FHS implantation. Aquino et al. [27], using panel data with a negative binomial response, reported a robust association between FHS coverage and infant mortality reduction from 1996 to 2004.

Hospital beds and other FHS team members are not found to be statistically significant in explaining infant mortality in the preferred specification. A high correlation was noted between PCPs and other FHS team members. However, the PCP coefficient was -6.40 after omitting the other FHS team members variable in column (9), showing no influence on its statistical significance and small influence on its magnitude. Previous studies for Brazil that use hospital beds and nurses density as independent variables to account for infant mortality have not reached a consensus. Macinko et al. [23] found both variables negatively associated with infant mortality, but only hospital beds was statistically significant. Sousa et al. [9] showed nurse density had an adverse and significant effect on infant mortality. By contrast, Macinko et al. [39] recorded that the hospital beds coefficient is positive and significant, but the study did not includes nurses.

The results show that municipalities with higher GDP per capita were associated with lower infant mortality rates. The association between income and health outcomes is well established in the literature. This relationship is found using different measurements of income, including GPD per capita [41]. Preston [42] argues that national income is probably the best indicator of living standards in a region, as well as representing a large range of factors that can influence mortality.

Kassouf [43] found that, in the same way as income, supplemental health insurance coverage is associated with better health status. Although The Brazilian health system—Unified Health System (SUS)—has universal access, around 24.4% of population was covered by supplemental health insurance in 2012 [44]. There are great inequalities in the use of health services in Brazil. Cambota & Rocha [45] showed that income, education and supplemental health insurance coverage contribute to increase the inequalities in these services. There are evidence that individuals who have supplemental health insurance visit a physician on average around 25% more than individual with no supplemental health insurance coverage; for hospital admissions the differential ranges from 8% to 15% [46]. We found a negative effect of supplemental health insurance coverage on infant mortality.

Our results are robust to including time trends and a range of important determinants of infant mortality rate such household facilities. Trash collection, piped water and available electricity are found relevant in accounting for changes in infant mortality rate in developing countries [47–51]. Electricity access facilitates more hygienic preparation of food and sterilization [52]. Piped water can cause a significant reduction in the infant mortality rate mainly by reducing the incidence of diarrhea [53]. Diarrhea is more prevalent in the developing countries due the lack of safe drinking water, sanitation and hygiene and it was responsible for 1.5 deaths among children under-5 worldwide in 2004 [54].

In additional tests, we included the *Bolsa Familia* Program (BFP) as a control variable. The literature has indicated the relevance of this conditional cash transfer program for infant/child mortality [55,56]. Our results remained consistent and showed small variations in the magnitude of the effect of PCPs after controlling for the BFP. However, the size of the BFP coefficient was sensitive to the control of time trends.

The effect of FHS PCPs was tested separately from the ‘traditional’ PCPs (S3 Appendix). The results showed that only the FHS PCPs was associated with lower infant mortality. The result confirms the relevance of the FHS for infant health, as reported in previous studies [23,27,39,40]. The coefficient of traditional PCPs (general practitioners, pediatricians and obstetrician-gynecologists) was not statistically significant, maybe because traditional physicians have increasingly been replaced by the FHS in recent years across Brazilians municipalities. The reduction of the traditional model of primary care, and expansion of FHS, has been based on a national strategy for reorganization of primary care [20].

In addition to the density and coverage of primary health care team, the literature has also indicated the relevance of consolidating primary care. There is evidence that municipalities with a consolidated FHS recorded a further reduction in infant mortality rates than municipalities without FHS coverage, incipient coverage and intermediate coverage [27]. This finding was also reported by Rasella, et al. [57] for neonatal, postneonatal and children mortality.

Finally, our extensive robustness checks including alternative model specifications and a placebo regression strengthen the validity of our findings. The study’s limitations include the quality of mortality data, which vary across region of Brazil. However, overall these additional estimations improves the results’ validity since we find similar results.

The above discussion highlighted that studies using data from earlier time periods produced results of a different magnitude but a similar direction of effect. Since 2012, when our data ended, there have been significant changes in both the economy and in PCP policy that could potentially lead to different effects than we have observed here. First, there was a significant economic downturn with a significant recession in 2015 and 2016. If this differentially affected municipalities, then the effect of PCP supply on infant mortality could be different post-2012. Second, alongside the economic crisis, fiscal austerity policies have recently been implemented in Brazil. These measures will reduce public expenditure as a percentage of GDP over 20 years, substantially affecting the healthcare system [58]. Studies have pointed out that the major impacts of this policy may be felt by the poorest and the most vulnerable [59]. In the long-term, child morbidity and mortality may also be considerably affected [60].

The Brazilian government introduced the More Doctors Program in 2013 and although evaluations of this program have reported a positive effect on some health outcomes [61], they found little evidence of an effect on infant mortality [62]. This could either be due to diminishing returns and/or the effects of the economic downturn on other factors that influence infant mortality. This highlights that the effectiveness of PCPs may change over time and may vary with economic cycles.

## Conclusions

Brazilian health policy has placed substantial importance on primary care in past decades, potentially providing lessons for other developing countries. The objective of this study has been to investigate infant mortality and the level of primary care physicians, including a range of important determinants of infant health. The results indicate a negative association between primary care physicians and infant mortality rates in Brazil. Moreover, a reduction of illiteracy among woman and a rise in the private health insurance coverage, GDP per capita, percentage of household with trash collection and piped water has also contributed to the decline of infant mortality.

Primary health care is regarded as a key component for building a strong healthcare system that ensures positive health outcomes, quality in healthcare and universal access. Brazil’s experience demonstrates that increases of primary care physician supply represents one strategy to reduce infant deaths and remains a potential instrument to achieve international goals related to child health.

## Supporting information

S1 Appendix Data availability. (DOCX)

S2 Appendix Estimation results for infant mortality using different estimation techniques. (DOCX)

S3 Appendix Estimation results for traditional and FHS physicians. (DOCX)

S4 Appendix Estimation results for external causes mortality. (DOCX)
