# Supplementary material for: Primary care physicians and infant mortality: Evidence from Brazil
Source: PLoS One. 2019 May 31;14(5):e0217614. doi: 10.1371/journal.pone.0217614 (PMC6544253; doi:10.1371/journal.pone.0217614)
Supplement: S1 Appendix — (DOCX) [file pone.0217614.s001.docx]

**S1 Appendix - Data Availability**

The data used in the paper are available in the Open Acess ICPS Data Repository. There are two data sets. The first is the initial dataset that was constructed after we downloaded the raw data from the publicly available websites below (<http://doi.org/10.3886/E108526V1>). The second dataset is the final data set we used to estimate the regression models (<http://doi.org/10.3886/E108527V1>).

The raw source data are publicly available from the websites below.

i) Infant mortality: *Óbitos por residência*. DATASUS/ Brazilian Ministry of Health. <http://tabnet.datasus.gov.br/cgi/deftohtm.exe?sim/cnv/inf10br.def>;

ii) Hospital beds: *Quantidade existente*. DATASUS/ Brazilian Ministry of Health. <http://tabnet.datasus.gov.br/cgi/deftohtm.exe?cnes/cnv/leiintbr.def>;

iii) Primary care physician and other FHS team: *PF – Professional*. CNES/DATASUS/Ministry of Health. <http://www2.datasus.gov.br/DATASUS/index.php?area=0901&item=1&acao=31&pad=31655>;

iv) Supplemental health insurance: *Assistência médica*. National Regulatory Agency for Private Health Insurance and Plans (ANS). <http://www.ans.gov.br/anstabnet/cgi-bin/dh?dados/tabnet_02.def>;

v) GDP: Brazilian Institute of Geography and Statistics (IBGE). <https://sidra.ibge.gov.br/tabela/5938>;

vi) Household facilities (trash collection, sewage, available electricity and access to piper water): *Microdados*. National Census Bureau. <https://www.ibge.gov.br/estatisticas-novoportal/sociais/saude/9663-censo-demografico-2000.html?=&t=microdados>;

vii) Female illiteracy: *RAIS Vínculo*. Annual List of Social Information (RAIS)/Ministry for Labor and Employment (MTE). Please note: this site (of the Ministry for Labor and Employment) requests a previous registration to access the data. It is free to register.

<http://bi.mte.gov.br/bgcaged/caged_rais_vinculo_id/caged_rais_vinculo_basico_tab.php>
