# Supplementary material for: Primary care physicians and infant mortality: Evidence from Brazil
Source: PLoS One. 2019 May 31;14(5):e0217614. doi: 10.1371/journal.pone.0217614 (PMC6544253; doi:10.1371/journal.pone.0217614)
Supplement: S2 Appendix — (DOCX) [file pone.0217614.s002.docx]

S2 Appendix - Estimation results for infant mortality using different estimation techniques

|  | Pooled OLS | Pooled OLS | Fixed Effects | Random Effects | Fixed Effects | Random Effects |
| --- | --- | --- | --- | --- | --- | --- |
|  |  |  |  |  |  |  |
| Infant Mortality_t-1_ | 0.110*** | 0.0861*** | -0.110*** | 0.110*** | -0.120*** | 0.0861*** |
|  | (0.009) | (0.009) | (0.008) | (0.011) | (0.008) | (0.011) |
| PC Physicians | -1.909*** | -1.293* | -2.254** | -1.909*** | 1.258 | -1.293* |
|  | (0.562) | (0.732) | (1.130) | (0.562) | (1.277) | (0.725) |
|  |  |  |  |  |  |  |
| Year | No | Yes | No | No | Yes | Yes |
| Additional controls | No | Yes | No | No | Yes | Yes |
| Observations | 38,938 | 38,938 | 38,938 | 38,938 | 38,938 | 38,938 |
| N of municipalities |  |  | 5,563 | 5,563 | 5,563 | 5,563 |
| F- test | 95.71*** | 107.73*** | 97.32*** |  | 36.17*** |  |
| Wald Chi2 |  |  |  | 111.44*** |  | 998.31*** |
| Hausman test |  |  | chi^2^(2) = 6924.90*** | | chi^2^(11) = 6608.80*** | |
| Standard errors in parentheses | |  |  |  |  |  |
| *** p<0.01, ** p<0.05, * p<0.1 | |  |  |  |  |  |
