# Supplementary material for: Primary care physicians and infant mortality: Evidence from Brazil
Source: PLoS One. 2019 May 31;14(5):e0217614. doi: 10.1371/journal.pone.0217614 (PMC6544253; doi:10.1371/journal.pone.0217614)
Supplement: S3 Appendix — (DOCX) [file pone.0217614.s003.docx]

S3 Appendix - Estimation results for traditional and FHS physicians

|  | System GMM |
| --- | --- |
|  |  |
| Infant Mortality_t-1_ | 0.0231** |
|  | (0.0108) |
| FHS Physicians | -10.50*** |
|  | (3.596) |
| Traditional Physicians | 0.413 |
|  | (3.278) |
|  |  |
| Year | Yes |
| Additional controls | Yes |
| Observations | 38,938 |
| N of municipalities | 5,563 |
| Instruments | 29 |
| Hansen/Sargan test | 16.81 |
| AR1 test | -25.02*** |
| AR2 test | 1.29 |
| Wald Chi2 | 855.46*** |
| Standard errors in parentheses. *** p<0.01, ** p<0.05, * p<0.1 | |
