# Supplementary material for: Primary care physicians and infant mortality: Evidence from Brazil
Source: PLoS One. 2019 May 31;14(5):e0217614. doi: 10.1371/journal.pone.0217614 (PMC6544253; doi:10.1371/journal.pone.0217614)
Supplement: S4 Appendix — (DOCX) [file pone.0217614.s004.docx]

S4 Appendix - Estimation results for external causes mortality

|  | System GMM |
| --- | --- |
|  |  |
| External Causes_t-1_ | 0.043 |
|  | (0.2497) |
| PC Physicians | 0. 0068 |
|  | (0. 0519) |
| Year | Yes |
| Additional controls | Yes |
| Observations | 38,941 |
| N of municipalities | 5,563 |
| Instruments | 23 |
| Hansen/Sargan test | 33.39*** |
| AR1 test | -2.12** |
| AR2 test | 0.09 |
| Wald Chi2 | 1540.09*** |
| Standard errors in parentheses. *** p<0.01, ** p<0.05, * p<0.1 | |
